# Early palmar plate fixation of distal radius fractures may benefit patients aged 50 years or older: a randomized trial comparing 2 different treatment protocols

**DOI:** 10.1080/17453674.2018.1561614

**Published:** 2019-01-23

**Authors:** Kai Sirniö, Juhana Leppilahti, Pasi Ohtonen, Tapio Flinkkilä

**Affiliations:** a Department of Surgery, Division of Orthopaedic and Trauma Surgery, Oulu University Hospital, Oulu;; b Department of Anesthesiology, Surgery, and Intensive Care, Oulu University Hospital, Oulu, Finland

## Abstract

Background and purpose — There is no consensus regarding optimal treatment of displaced distal radius fractures (DRFs). We compared the results of 2 treatment protocols: early palmar plating vs. primary nonoperative treatment of displaced DRFs.

Patients and methods — We performed a prospective randomized controlled study including 80 patients aged ≥ 50 years with dorsally displaced DRFs, excluding AO type C3 fractures. Patients were randomized to undergo either immediate surgery with palmar plating (n = 38), or initial nonoperative treatment (n = 42) after successful closed reduction in both groups. Delayed surgery was performed in nonoperatively treated patients showing early loss of alignment (n = 16). The primary outcome measure was Disabilities of the Arm, Shoulder, and Hand (DASH) score.

Results — Mean DASH scores at 24 months in the early surgery group were 7.9 vs. 14 in the initial nonoperative group (difference between means 6, 95% CI 0.1–11, p = 0.05). Delayed operation was performed on 16/42 of patients due to secondary displacement in the initial nonoperative group. In “as treated” analysis, DASH scores were 7 in the early surgery group, 13 in the nonoperative group, and 17 after delayed surgery (p = 0.02). The difference in DASH scores between early and delayed surgery was 9 points (CI 0.3–19, p = 0.02)

Interpretation — Treatment of DRFs with early palmar plating resulted in better 2-year functional outcomes for ≥50-year-old patients compared with a primary nonoperative treatment protocol. Delayed surgery in case of secondary displacement was not beneficial in terms of function.

As palmar plating of distal radius fractures (DRFs) became more popular, the incidence of operative treatment increased, particularly among older women (Chung et al. 2009, Mattila et al. 2011, Mellstrand-Navarro et al. 2014).

Percutaneous Kirschner wire (K-wire) fixation and ORIF with palmar plating are the most used fixation methods of displaced DRFs. The previous DRAFFT study showed no difference in functional results at 12 months between percutaneous K-wire fixation and volar plating in adults (Costa et al. 2014). Palmar fixed-angle plates enable near-anatomic reduction and stable fixation, creating optimal conditions for healing even in osteoporotic bone (Orbay and Fernandez 2004, Figl et al. 2010). However, while palmar plate fixation of DRF achieves stability and good radiographical results (Orbay and Fernandez 2004, Rozental and Blazar 2006), the relationship between radiographic reduction and outcome is not yet confirmed, particularly in elderly patients (Grewal and MacDermid 2007, Diaz-Garcia et al. 2011). Considering that nonoperative treatment has been in general the preferred treatment method, it is of interest to compare results of palmar plating and nonoperative treatment of DRFs. Only a few randomized controlled trials have compared mid-term results of palmar plating and nonoperative treatment of DRFs in populations predominantly including elderly patients, with no significant benefit of plating observed (Arora et al. 2011, Bartl et al. 2014). In contrast, a recent study of Martinez-Mendez et al. (2018) showed significantly better functional results in patients older than 60 years treated with palmar plating compared with cast treatment of DRF.

After closed reduction of the displaced DRF in our department, fracture alignment is routinely evaluated at 1-week and 2-week follow-up visits. When malalignment reaches a specific threshold (> 10° of dorsal angulation, < 15° of radial inclination, or > 2° millimeters ulnar positive variance), patients are offered surgery. Loss of alignment within 2 weeks after closed reduction of DRF is common (Mackenney et al. 2006), and it is challenging to decide between operative and nonoperative treatment at this early stage. Early operative treatment may lead to more predictable radiographic and patient-rated outcomes, with fewer outpatient visits.

In the present study, our main goal was to compare functional and radiographic results in patients of ≥ 50 years of age with dorsally displaced DRFs who underwent treatment with 2 different protocols: early palmar plating vs. primary nonoperative (control group) treatment. Our primary hypothesis was that the functional results at 24 months would be superior in the early surgery group compared with the control group. Our secondary aim was to compare the patient satisfaction, radiographic results, complications, and secondary operation rates between study groups.

## Patients and methods

We conducted a prospective randomized controlled single-center trial at Oulu University Hospital (Department of Surgery, Division of Orthopaedic and Trauma Surgery, Oulu, Finland). 80 patients of ≥ 50 years of age were recruited from the catchment area of our hospital district between November 2008 and January 2014.

### Patients

**Figure 1.**
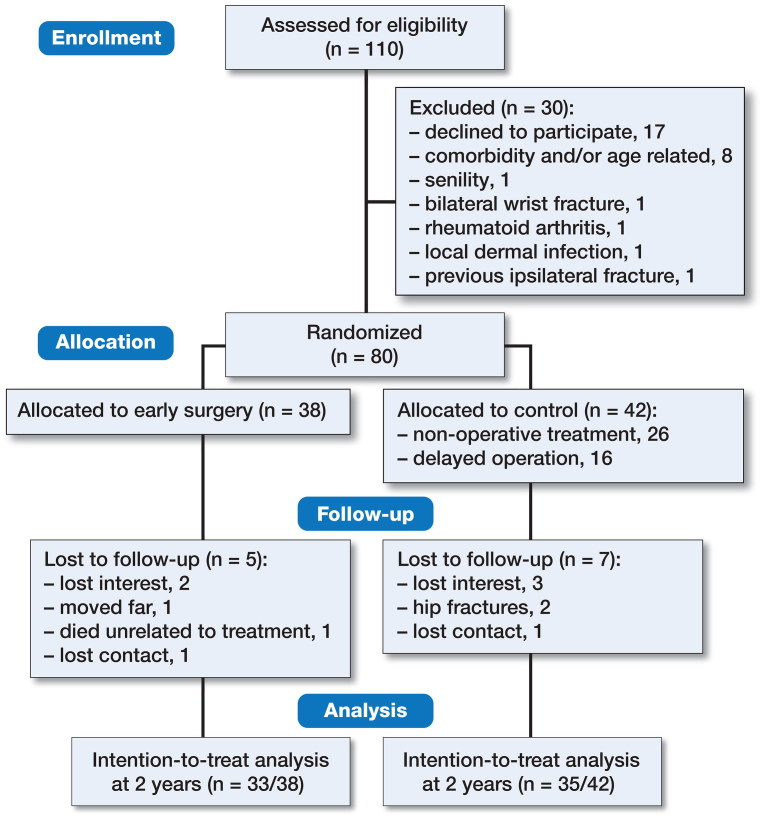
Flow diagram.

Table 1 presents the criteria for inclusion and exclusion. All fractures were in both groups initially treated via closed reduction under hematoma block and acceptable radiographic reduction was achieved in all cases. The wrist was immobilized with a short-arm cast at the emergency unit. 110 patients were assessed for eligibility, of whom 30 were excluded (Figure 1). The remaining 80 patients were randomized into 1 of 2 study protocol groups: early surgery with palmar plating (n = 38) or primary nonoperative treatment (n = 42).

**Table 1.** Inclusion and exclusion criteria

**Inclusion criteria**
Displaced DRF (AO/OTA 23 type A2/A3 and C1/C2)
Duration of < 1 week from primary injury
Acceptable closed reduction achieved:
– dorsal angulation ≤ 10°
– radial inclination ≥ 15°
– ulnar variance < –3 mm
– articular step-off ≤ 2 mm
**Exclusion criteria**
Patients < 50 years old
Acceptable closed reduction not achieved
(see inclusion criteria)
Bilateral/open fractures
Fractures with neurovascular compromise
Previous ipsilateral DRF
Inflammatory joint disease
Radiocarpal joint degeneration
Limited cooperation or major comorbidity not allowing to
operate
Major concomitant fracture necessitating any operation

### Intervention

Within 1 week after injury, patients underwent palmar plating of DRF applying a standard surgical technique. The distal part of the radius was exposed using a modified Henry’s approach. Fracture reduction was achieved by open manipulation, and the fracture was stabilized using a palmar fixed-angle plate (Aculoc or Aculoc 2; Acumed, Hillsboro, OR, USA) with proximal 3.5-mm locking screws and distal 2.3-mm locking cortical pegs. A dorsal plaster cast for pain relief was applied for 10 days, after which the patients received formal instructions for active mobilization of the wrist. All procedures were performed by experienced surgeons familiar with the operative technique being used.

### Control

Patients allocated to the control group underwent closed reduction in the emergency department, and were then immobilized in a below-elbow arm cast. They were scheduled to attend follow-up visits at 1 and 2 weeks after treatment. If the reduction met the acceptable criteria, immobilization was continued for 6 weeks, after which the cast was removed and active mobilization of wrist was started. Operative treatment with a palmar plate was offered when there was greater than 10° dorsal angulation of the articular surface on the lateral radiograph, less than 15° of radial inclination or greater than 2 mm ulnar positive variance on the posteroanterior radiograph within 2 weeks after injury. If patient declined surgery, non-operative treatment was continued until fracture union. In the case of delayed surgery in the control group, the postoperative follow-up and rehabilitation program was identical to that of the intervention group.

### Outcome *measures*

The primary outcome measure was the Disabilities of the Arm, Shoulder, and Hand (DASH) score (Hudak et al. 1996, Aro et al. 2008) at 2 years. Secondary outcome measures included range of motion (ROM), grip strength, subjective assessment of wrist function, radiological results, complications, and rate of re-operations. Follow-up visits for both groups were scheduled at 3, 6, 12, and 24 months.

### Function

At each follow-up visit, the patients completed DASH questionnaires. All objective functional measurements were performed by a physiotherapist not involved in patient care. Dorsal and volar flexion and ulnar and radial deviation were measured using a manual goniometer. Pronation and supination were measured using a Myrin compass goniometer (Follo Futura AS, Ås, Norway). Grip strength (kg) was measured using a calibrated Hydraulic Hand Dynamometer Model SH5001 (Saehan Corporation, Changwon, South Korea). The best result of 3 attempts was used for analysis. Patients were also asked to subjectively grade the function of their injured wrist as poor, fair, good, or excellent using the uninjured side as reference.

### Radiography

In both treatment groups standard posteroanterior (PA) and lateral wrist radiographs were taken immediately after primary injury and after closed reduction of the fracture. In the control group, radiographs were taken at 1, 2, 6, and 12 weeks and at 6, 12, and 24 months after injury. In the operative treatment group, radiographs were taken at postoperative day 1 and at 6 weeks, 12 months, and 24 months after the injury. The uninjured side was radiographed at 6 weeks after injury. KS made digital measurements from the radiographs. Fracture types were classified according to AO classification, using the main types A and C to maintain reproducibility (Flinkkilä et al. 1998). Dorsal angulation was measured from lateral radiographs and radial inclination from posteroanterior radiographs. Ulnar variance (in mm) was measured from PA radiographs with reference to the uninjured side.

### Adverse effects

An adverse event was defined as any unfavorable or unintended sign that could affect the results or that necessitated secondary surgery. Malunion was defined as dorsal angulation of > 15° and radial inclination of < 15°. Radiocarpal osteoarthritis was assessed and graded according to Knirk and Jupiter (1986). Re-operation was defined as secondary surgery related to the primary injury or operation.

### Sample size

Sample size was calculated using the DASH score, assuming a clinically relevant 15-point difference between treatments and standard deviation (SD) of 22, based on previous studies (Gummesson et al. 2003, Anzarut et al. 2004, Rozental and Blazar 2006). The calculation indicated a need for 35 patients per group (SD = 22, α = 0.05, power = 0.80), with an estimated 10% dropout rate. We decided to include 80 patients in the study.

### Randomization

Patients were randomly allocated into study groups based on a computer-generated list. Randomization was performed in blocks, with block sizes randomly varying between 4, 6, 8, and 12. Separate lists were created for age groups of < 65 and ≥ 65 years, and for type A and C fractures. Randomization lists were sealed into numbered opaque envelopes. After confirmation of patient eligibility and obtaining the patient’s written informed consent, the treating surgeon opened a numbered envelope revealing the method of treatment.

### Statistics

The patients were analyzed primarily on an intention-to-treat basis and secondarily “as treated.” Missing data in our primary outcome variable DASH at 24 months’ follow-up of randomized patients were imputed using a multiple imputation (MI) method. The missing data pattern was non-monotone and therefore we used MI with fully conditional specification using variables DASH (3, 6, and 12 months) and age, fracture type, and randomization group to model data for DASH at 24 months. 50 different data sets were created and the pooled result is presented. Summary measurements are presented as mean (SD) or as median with 25th and 75th percentiles. Comparisons between study groups were performed using Student’s t-test or the Mann–Whitney U-test for continuous variables, and by the chi-square or Fisher’s exact test for categorical variables. The Kruskal–Wallis test was used to compare DASH results at the 2-year follow-up between the operative, nonoperative to the end, and delayed operation groups. If p < 0.05 according to Kruskal–Wallis test then Mann–Whitney U-test was used for comparisons between the 2 groups. Repeatedly measured data were analyzed using a linear mixed model (LMM) assuming patients as random effects. The covariance pattern was chosen according to Akaike’s information criteria. The p-values reported for LMM are p_time_ for the change over time, p_group_ for average treatment difference, and p_time x group_ for interaction between time and treatment. The results of between-group comparisons are presented as the difference between means and 95% confidence interval (CI). Comparisons of functional outcome scores between age groups were preplanned subgroup analyses, while comparisons between early surgery, delayed surgery, and nonoperative treatment (“as treated”) were decided post hoc.

### Ethics, registration, funding and potential conflicts of interest

All patients gave written informed consent, and the study was approved by the Oulu University Hospital Ethics Committee (number EETTMK: 143/2007) and registered at Clinicaltrials.gov (NCT02990052). The authors received no financial support for the research, authorship, and/or publication of this article and the authors declared no potential conflicts of interest with respect to the research, authorship, and/or publication of this article.

## Results

The main study groups had comparable baseline demographic characteristics (Table 2). 16 patients in the control group underwent delayed operation at 1 to 3 weeks after the initial trauma due to early loss of reduction (Figure 1). 2 patients in the control group declined delayed surgery and were treated nonoperatively.

**Table 2.** Demographics. Values are number of patients unless otherwise indicated

Factor	Early surgery group	Control group
	(n = 38)	(n = 42)
Age in years, mean (range)	62 (50–79)	64 (50–82)
Age ratio, < 65/≥ 65 years	23/15	23/19
Sex, female/male	37/1	39/3
Dominant hand involved	15	17
AO classification		
A	23	25
C	15	17

### Function

**Figure 2.**
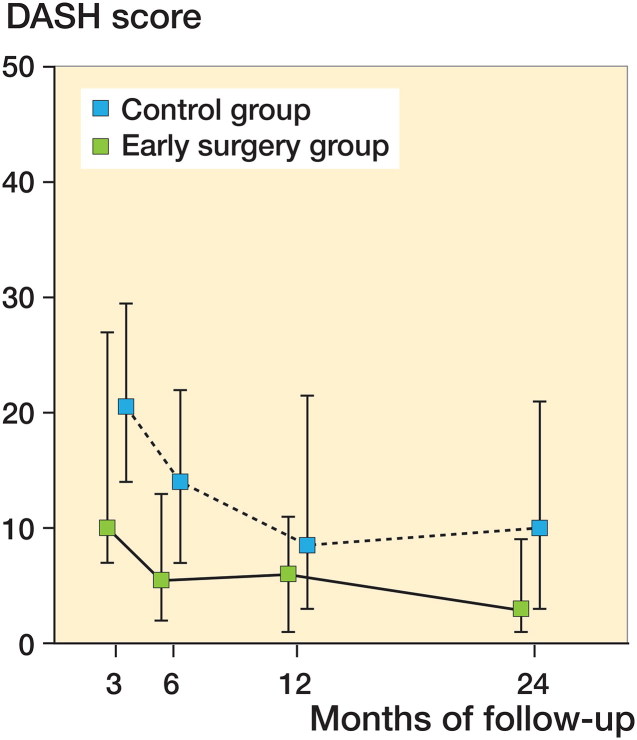
DASH scores for main study groups at follow-up points. Scores are presented as median with 25th and 75th percentiles. The p-values were determined according to a linear mixed model (LMM): p_time_ < 0.001, p_group_ = 0.04, and p_time x group_ = 0.6.

Mean DASH scores at the 2-year follow-up differed statistically significantly between study groups, favoring early operation: 7.2 vs. 14.4, p = 0.005 (difference between means, −7; CI −13 to −1.5) (Figure 2). According to MI analysis the mean difference reduced being –6 points (8 vs. 14; CI –11 to –0.12, p = 0.05). At the 2-year follow-up, statistically significant differences favoring early surgery were also detected in flexion (71° vs. 64°; p = 0.002; difference between means, 7°; CI 3–12) and ulnar deviation (28° vs. 25°; p = 0.009; difference between means, 3°; CI 0.9–6). In terms of ROM, only the recovery rate of extension was faster in the early surgery group. Grip strength at 2-year follow-up was comparable between study groups (Table 3, Supplementary data). Wrist function was self-assessed to be excellent or good by 30/33 patients in the early surgery group compared with 23/35 patients in the control group (p = 0.01).

In analysis limited to patients of ≥ 65 years of age, DASH scores at the 2-year follow-up did not differ statistically significantly between the early surgery and control groups: 11 vs. 17 (difference between means, −6; CI −18 to 4; p = 0.2). For the patients below 65 years of age the mean DASH for early surgery and control groups was 6 vs. 11 (difference between means, −5; CI −12 to –1; p = 0.01).

In “as treated” analysis, the mean DASH scores at the 2-year follow-up were 7 (SD 10) in the early surgery group, 13 (SD 12) in the nonoperative to the end group, and 17 (SD 16) in the delayed operation group (p = 0.02). A statistical, and probably also clinically significant difference between early and delayed surgery groups was found (difference between means, –9; CI –19 to –0.3; p = 0.02).

### Radiography

All radiographic parameters were statistically significantly better in the early surgery group compared with the control group (Table 4, Supplementary data). Of the 42 patients in the control group, 18 exhibited secondary loss of reduction at the 1- or 2-week follow-up visits, and 16 of these patients underwent delayed operation at between 1 and 3 weeks after primary injury. In the conversion group mean dorsal angulation was −1.1° (SD 5), radial inclination 24° (SD 4), and ulnar variance −0.6 mm (SD 1.2). Loss of acceptable alignment after 2 weeks’ follow-up visits was observed in 14/42 patients in the control group at final follow-up.

### Complications and secondary operations

1 case of carpal tunnel syndrome, 1 patient with flexor tenosynovitis, and 1 case of post-traumatic radiocarpal arthritis was noted in the early operation group. Only the patient with carpal tunnel syndrome required secondary operation. In the control group 4 patients with a carpal tunnel syndrome and 1 case of grade I post-traumatic radiocarpal osteoarthritis were noted. 3 patients in the control group had secondary operation, 2 with carpal tunnel syndrome and 1 with malunion.

## Discussion

Our results showed that DASH score at 2 years favored early surgery over initial nonoperative treatment of displaced DRFs. Wrist flexion and ulnar deviation at 24 months favored early surgery, but other between-group differences in ROM and grip strength after 6 months were minor. Surgery resulted in earlier recovery of wrist extension than achieved with primary nonoperative treatment. Compared with the control group, the early surgery group showed better patient satisfaction with superior radiographic results and fewer complications and secondary operations. Delayed operation in cases of secondary displacement after initial nonoperative treatment did not provide comparable results to early surgery in terms of DASH score.

Arora et al. (2011) performed an RCT comparing palmar plating and nonoperative treatment for unstable DRFs in patients ≥ 65 years old, and found no statistically or clinically significant between-group differences in DASH score or ROM at 12 months. Their study differed from ours, as we included only fractures that were acceptably reduced. Nevertheless, our findings regarding functional results in an elderly cohort support those of Arora et al. (2011). Additionally, Bartl et al. (2014) performed a multi-center RCT comparing open reduction and internal fixation with cast treatment of primary unstable intra-articular DRFs in patients of ≥ 65 years of age with 12 months of follow-up, and reported no significant between-group differences in DASH scores or ROM.

In the control group, almost half of the fractures lost alignment within 2 weeks after acceptable primary reduction, in line with previous findings (Mackenney et al. 2006, Bartl et al. 2014, Martinez-Mendez et al. 2018). Yamashita et al. (2015) performed a retrospective study of extra-articular DRFs, and found no difference in functional results between early and delayed fixation at 1 year of follow-up. Our study included intra-articular fractures and had a longer follow-up time, possibly explaining the difference. Weil et al. (2014) performed a retrospective study, including mostly type C fractures, and showed statistically worse Quick-DASH scores at one year in the delayed surgery group (operated > 21 days after injury) compared with the historical cohort of primarily operated patients. In our study, delayed operation was performed in more than one-third of patients in the control group because of secondary displacement, with inferior results compared with early surgery. The data suggest that the common treatment protocol of initial closed reduction and secondary surgery of partially healed fracture after secondary displacement is not optimal. Hence the treatment method should probably be decided at an early stage after injury. Also, late instability after 2 weeks in our study population was common, comprising one-third of nonoperatively treated patients. This supports previous findings (Mackenney et al. 2006, Wadsten et al. 2018). It could be useful to apply treatment algorithms based on predictors of fracture instability (Mackenney et al. 2006, Walenkamp et al. 2016), but the value in clinical practice is not yet proven.

Although the functional scores favored the early surgery group, the higher patient satisfaction in the early operation group was unexpected and may have been due to the higher rate of malalignment and secondary operations in the control group. Unstable fractures that lose alignment had inferior results in general compared with stable ones in control group, which could explain this difference. Although DASH and patient-rated wrist evaluation (PRWE) are valid outcome measures, they probably cannot capture all impairments, as shown in this and previous studies (Plant et al. 2017).

Our study has several strengths. The RCT design of this study comparing 2 commonly used treatment protocols generated high-quality evidence regarding treatment of this common fracture. The investigated fracture types are the most challenging in terms of decision-making. Both groups had similar baseline demographics, and the catchment of patients at the 1- and 2-year follow-up points was sufficient. Moreover, one experienced surgeon treated most of the cases.

This study also has several weaknesses. Due to limited resources we could screen only a proportion of all DRF patients treated at the emergency clinic during the study period. There was some patient dropout during the study and in accordance with our primary study protocol we also operated on a few stable fractures in the early surgery group, which may have biased the results. Type C3 fractures were not included, which we considered to be unsuitable for nonoperative treatment. Therefore results cannot be generalized to all fracture types. Considering our primary intention and sample size analysis to compare 2 main study groups, caution should be used when analyzing the results of subgroups of the study.

In summary, early surgery after primary closed reduction of DRF could be recommended to physically active patients, if secondary displacement is imminent. High displacement rate after primary nonoperative treatment was observed and delayed surgery in these cases was not beneficial in terms of function when comparing with early surgery. The decision between surgery and nonoperative treatment should be made at a very early stage, and delayed operation avoided in cases of secondary displacement of DRFs in elderly people.

## Supplementary Material

Supplemental Material
