# Supplementary material for: Early palmar plate fixation of distal radius fractures may benefit patients aged 50 years or older: a randomized trial comparing 2 different treatment protocols
Source: Acta Orthop. 2019 Jan 23;90(2):123–8. doi: 10.1080/17453674.2018.1561614 (PMC6461076; doi:10.1080/17453674.2018.1561614)
Supplement: Supplemental Material [file IORT_A_1561614_SM5777.pdf]

Supplementary data

Table 3. ROM and grip strength. Values are given as mean (SD)

Factor	Early surgery group	Control group	p-values [a] p_{time}	p_{group}	$p_{time\ x\ group}$
Extension (°):			< 0.001	0.2	0.04
3 months	62 (10)	56 (12)			
6 months	64 (8)	65 (11)			
12 months	68 (8)	66 (9)			
24 months	69 (6)	68 (7)	(diff. 1°, 95% CI –2 to 4)		
Flexion (°):			< 0.001	< 0.001	0.2
3 months	58 (12)	51 (12)			
6 months	64 (8)	55 (11)			
12 months	66 (8)	61 (11)			
24 months	71 (7)	64 (11)	(diff. 7°, CI 3–12)		
Radial deviation (°):			0.02	0.7	0.5
3 months	19 (5)	20 (9)			
6 months	21 (4)	21 (5)			
12 months	22 (5)	21 (4)			
24 months	22 (6)	22 (5)	(diff. 0°, CI –3 to 3)		
Ulnar deviation (°):			<0.001	<0.001	0.3
3 months	24 (6)	20 (7)			
6 months	27 (6)	21 (6)			
12 months	28 (6)	22 (6)			
24 months	28 (5)	25 (6)	(diff. 3°, CI 1–6)		
Pronation (°):			< 0.001	0.8	0.8
3 months	83 (8)	83 (12)			
6 months	85 (8)	87 (7)			
12 months	87 (5)	86 (8)			
24 months	88 (5)	88 (6)	(diff. 0°, CI –2 to 3)		
Supination (°):			< 0.001	0.05	0.2
3 months	78 (13)	73 (17)			
6 months	85 (6)	79 (12)			
12 months	85 (7)	83 (10)			
24 months	88 (5)	84 (10)	(diff. 3°, CI –1 to 7)		
Grip strength (kg):			< 0.001	0.06	0.2
3 months	21 (5)	18 (5)			
6 months	25 (5)	22 (5)			
12 months	26 (5)	25 (5)			
24 months	27 (5)	26 (7)	(diff. 1 kg, CI –2 to 4)		

[a] The presented p-values were determined according to a linear mixed model (LMM). Differences between means and 95% confidence intervals are represented only at 24 months.

Table 4. Radiographic results. Values are given as mean (SD)

Factor	Early surgery group	Control group	p-values [a] p_{time}	p_{group}	$p_{time\ x\ group}$
Dorsal angulation (°)			< 0.001	0.003	< 0.001
Post-injury	26 (9)	22 (8)			
Post-reduction	3 (5)	2 (6)			
Postoperative	–5 (3)				
At 24 months	–3 (4)	3 (9)			
Radial inclination (°):			< 0.001	0.01	< 0.001
Post-injury	17 (4)	18 (5)			
Post-reduction	20 (4)	21 (5)			
Postoperative	23 (3)				
At 24 months	24 (4)	21 (8)			
Ulnar variance (mm):			< 0.001	0.002	< 0.001
Post-injury	–3.3 (1.7)	–2.7 (1.7)			
Post-reduction	–1.5 (1.0)	–1.2 (1.1)			
Postoperative	–0.2 (0.5)				
At 24 months	–0.7 (0.9)	–2.0 (1.7)			

Immediate postoperative values are indicated only for patients in the early surgery group.
